# Evaluation of energy expenditure in forward and backward movements performed by soccer referees

**DOI:** 10.1590/1414-431X20155061

**Published:** 2016-04-12

**Authors:** M.R. Paes, R. Fernandez

**Affiliations:** Departamento de Fisiologia, Setor de Ciências Biológicas, Universidade Federal do Paraná, Curitiba, PR, Brasil

**Keywords:** Referee, Football (soccer), Energy expenditure, Maximal oxygen uptake (VO_2max_)

## Abstract

The aim of this study was to measure the energy expenditure for locomotor activities usually performed by soccer referees during a match (walking, jogging, and running) under laboratory conditions, and to compare forward with backward movements. The sample was composed by 10 male soccer referees, age 29±7.8 years, body mass 77.5±6.2 kg, stature 1.78±0.07 m and professional experience of 7.33±4.92 years. Referees were evaluated on two separate occasions. On the first day, maximal oxygen uptake (VO_2max_) was determined by a maximal treadmill test, and on the second day, the oxygen consumption was determined in different speeds of forward and backward movements. The mean VO_2max_ was 41.20±3.60 mL·kg^-1^·min^-1^ and the mean heart rate achieved in the last stage of the test was 190.5±7.9 bpm. When results of forward and backward movements were compared at 1.62 m/s (walking speed), we found significant differences in VO_2_, in metabolic equivalents, and in kcal. However, the same parameters in forward and backward movements at jogging velocities (2.46 m/s) were not significantly different, showing that these motor activities have similar intensity. Backward movements at velocities equivalent to walking and jogging are moderate-intensity activities, with energy expenditure less than 9 kcal. Energy expenditure was overestimated by at least 35% when calculated by mathematical equations. In summary, we observed that backward movements are not high-intensity activities as has been commonly reported, and when calculated using equations available in the literature, energy expenditure was overestimated compared to the values obtained by indirect calorimetry.

## Introduction

Body weight can affect, either positively or negatively, the physical performance of an athlete. Therefore, weight control is extremely necessary for any person who is involved in a professional sport. The most effective body weight control is by maintaining a balance between food intake and energy expenditure (1). There are many factors that determine the daily energy requirements of athletes, including the type of physical activity that they perform (2). The energy expenditure caused by training depends on the amount and the intensity with which the movements are performed (3). Thus, determining the energy expenditure of an athlete during sports and training has great importance. However, while this parameter has been well evaluated in soccer players, it has not been so well explored in soccer referees. Although soccer players and referees are exposed to identical environmental conditions during a match, each plays a different role involving specific physical and cognitive demands (4,5). During a soccer match, the referee changes motion activity every 4-6 seconds, equating to more than 1200 different activities. Of these, almost half are low-intensity activities (standing, walking, jogging) and around 10% are of high intensity (running and sprinting) (6). These indicate that soccer refereeing is a highly intermittent exercise, in which the aerobic energy production accounts for approximately 90% of total energy consumption (7). The frequency of high intensity activities is reduced in the second period of the match, an event that coincides with the increase of the mean distance from infringements (8). These observations reinforce the idea of fatigue at the end of the match, and the possible negative effects on the referee's decision-making process. Presently, it is not clear if improving exercise capacity can decrease the probability of incurring judgment errors during the match.

Numerous studies have investigated the maximum oxygen consumption of soccer players during training and matches (9 10 11), but only one study has been conducted to determine the energy expenditure during lateral and backward movements (12). In this study, Reilly and Bowen (12) found significantly higher energy expenditure during backward movement at 9 km/h when compared to forward movement at the same velocity. Based on the mean energy expenditure found for backward movements (17.06 kcal) this activity was classified as of high intensity, concluding that such movements call for great metabolic demand and elevated cardiovascular response. However, there is no study that investigated the energy expenditure during backward movements in soccer referees. Da Silva et al. (13) have investigated the energy expenditure, reported as kilocalories (kcal), and metabolic equivalents of task (METs) of referees during the motor activities performed in official matches. A major limitation of these studies was that energy expenditure was predicted based on mathematical equations instead of direct measurements, such as indirect calorimetry (with gas exchange analysis). Thus, it is necessary to directly determine oxygen consumption in soccer referees during each motor activity (including backward movements) by using an open circuit spirometry.

Therefore, the aim of this study was to measure the energy expenditure for each locomotor activity performed by soccer referees during a simulated-match (walking, jogging, and running) under laboratory conditions, and to compare forward with backward movements.

## Material and Methods

### Participants and ethical procedures

The sample was composed by 10 male volunteer soccer referees accredited by the Paranaense Soccer Federation (FPF), with mean age of 29±7.8 years and a professional experience of 7.33±4.92 years. The participants had mean body mass of 77.5±6.2 kg, stature of 1.78±0.07 m, body mass index of 24.07±1.69 kg/m^2^, and body fat percentage of 19.9±2.1. Most of these characteristics were similar to other groups of official soccer referees reported in previous studies (4,14). Through a contact with the FPF, a list with all referees from the federation was obtained. Referees were randomly contacted by telephone and a brief explanation about the study was given. Volunteers who agreed to participate were informed verbally and by a written form about the nature and demands of the study, as well as about eventual health risks. They were also informed that they could withdraw from the study at any time. Written consent was obtained from each individual. Care was taken to ensure that subjects maintained their normal training and professional routines during the experimental period. All subjects were approved to work in official matches, during official physical tests and medical evaluation conducted by the FPF. The project was approved (Record #1076.11.03) by the Ethics Committee of the Hospital Universitário, Universidade Federal do Paraná, and it followed the 96/1996 resolution from the Brazilian National Council of Health.

### Experimental design

Referees were evaluated on two separate days. The first test performed was maximal oxygen uptake (VO_2max_) and the second test included evaluation of forward and backward movements. The second test was performed within at least 24 h after the VO_2max_ test. The agenda of all individuals was checked to verify that they were not refereeing any match or performing any strenuous effort during test periods. All tests were performed between 2:00 and 4:00 pm. Referees performed a familiarization session in a treadmill prior to testing, including backward movements. Five minutes of moderate intensity exercise (8 km/h) was used as warm-up, with the test beginning after 5 min of passive recovery.

During the first testing session, the VO_2max_ and velocity associated *v*VO_2max_ were obtained with the maximal incremental test at an initial velocity of 8 km/h incremented by 1 km/h every minute until exhaustion. Strong encouragement was given for subjects to achieve maximal intensity. Treadmill gradient was set at 1% (15). VO_2max_ was considered as the highest average consumption of O_2_ during the last 30 s of exercise, with at least two of the following criteria being obtained: Heart rate (HR) >maximal age-predicted HR (220 - age); respiratory gas exchange ratio (RER=carbon dioxide production (VCO_2_)/VO_2_) of 1.04 or higher; and a VO_2_ plateau (variation of <2.1 mL·kg^-1^·min^-1^ between the last two exercise stages). *v*VO_2max_ was considered as the lowest running velocity achieved at VO_2max_. The 1% gradient was used to favor the comparison among others studies (12,16) and to follow the Fédération Internationale de Football Association (17) recommendations that the playing field must be absolutely smooth and leveled. In the second testing session the oxygen consumption was determined during forward and backward movements. During the forward movement the oxygen consumption was determined in the following speeds: walking speed of 1.62 m/s (6 km/h); jogging at 2.46 m/s (9 km/h); running at 3.16 m/s (11 km/h); and sprinting at 5.08 m/s (18 km/h). These velocities were similar to those previously described in a study with Brazilian soccer referees (13). Subjects performed each movement for 3 min, with a break of 5 min between velocities. The average VO_2_ was measured during the last 30 s of exercise. However, we observed that VO_2max_ was obtained with speeds lower than 5.08 m/s, thus the energetic expenditure at that speed was evaluated through the anaerobic capacity (18). Backward movements were performed only at speeds of 1.62 m/s (6 km/h), and 2.46 m/s (9 km/h). This was done to simulate the situation found in the field during a game, where referees perform backward movements with the game stopped (no ball movements). If they need to move faster, referees usually turn and run forward. Although backward motion is not commonly performed in this equipment (treadmill), a previous study that verified the energetic expenditure during backward movement rated soccer players in the same condition (12).

The tests were performed on a motorized treadmill (Imbramed ATL, Inbrasport, Brazil). During all sessions, HR was continuously monitored using a Polar Heart Rate Monitor (Polar Electro Oy, Finland) and recorded beat-by-beat (R-R intervals) by an electrocardiography channel of a True-One system (TrueOne^¯^ 2400, Parvo Medics, USA). The energetic expenditure in kcal and METs were also obtained simultaneously and continuously through the consumption of oxygen and the individual parameters using the True System-One. Oxygen consumption and energetic expenditure in kcal were also estimated by mathematical equations, as previously described (13).

Pulmonary gas exchange was measured breath-by-breath in all sessions by determining O_2_ and CO_2_ concentrations, and ventilation, to calculate VO_2_ using a metabolic gas analysis system (True-One 2400, ParvoMedics). The gas analyzer was calibrated immediately before and verified after each test using a certified gravimetrically determined gas mixture, while the ventilometer was calibrated pre-exercise and verified post-exercise using a 3-L syringe, in accordance with the manufacturer's instruction (19). Following removal of outliers to exclude discrepant breaths, breath-by-breath VO_2_ data were interpolated to give 1s values, and cleaned using a rolling average analysis (30 s) (OriginPro 7.0, OriginLab Corporation, USA) to enhance the underlying VO_2_ response characteristics. Room temperature and humidity (mean 26±1.41°C and 46.5±6.0%, respectively) were controlled using an apparatus available with the metabolic analyzer used in the study (TrueOne^¯^ 2400, Parvo Medics).

The net efficiency was calculated according to Powers and Howley (2), in which Work is divided by consumed Energy (in kilojoules), multiplied by one hundred (Work ÷ Energy consumed × 100).

### Statistical analysis

Results are reported as means±SD. Normality was assessed using the Shapiro-Wilk test. Statistical comparisons among multiple means were made by repeated-measures one-way ANOVA followed by the Tukey multiple comparisons test. Energetic expenditure and net efficiency comparisons between forward and backward movements were determining using two-tailed Student's paired *t*-test. The statistical software used was GraphPad Prism, version 5.0 for Windows (GraphPad Inc., USA). Values for P<0.05 were considered to be statistically significant.

## Results

The mean VO_2max_ was 41.20±3.60 mL·kg^-1^·min^-1^ and the mean HR in the last stage of the test was 190.5±7.9 bpm. The energetic expenditure, measured in METs and in kilocalories (kcal), observed in the last stage of the test was 11.80±1.03 and 18.08±6.38, respectively.

**Figure 1:**
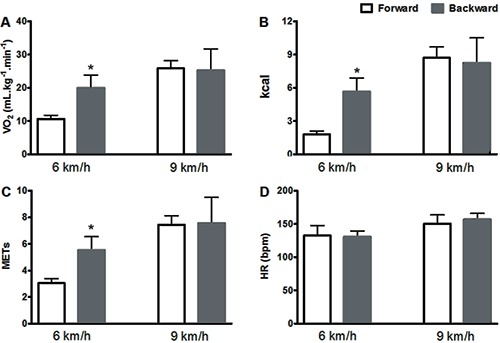
Energy expenditure in forward and backward movements at walking (6 km/h) and jogging (9 km/h) speeds. *A*, oxygen uptake (VO_2_); *B*, kilocalories (kcal); *C*, metabolic equivalent (METs); *D*, heart rate (HR). *P=0.001 *vs* forward movements (Student's paired *t*-test).

The energetic expenditure obtained at walking, jogging and running velocities was separately analyzed. In this aspect, the data were divided into forward and backward movements (Table 1). The repeated measures ANOVA test showed significant differences for VO_2_, HR, METs and kcal between all speeds in forward movements (Table 1). When we compared the results of forward and backward walking, we found significant differences in VO_2_, METs and kcal (Table 1). These results show that walking in backward movements is more intense than in forward movements at the same speed (Figure 1). However, the same parameters in forward and backward movements at jogging velocities were not significantly different, showing that these motor activities have similar intensity (Table 1, Figure 1). We also found that backward movements at velocities equivalent to walking and jogging are moderate-intensity activities, with energy expenditure lower than 9 kcal (Table 1). When oxygen consumption and energetic expenditure in kcal were calculated by mathematical equations we obtained values that were significantly higher to those directly determined by ergospirometry (Table 1). The only exception was with backward movements at walking velocity.

**Table:**
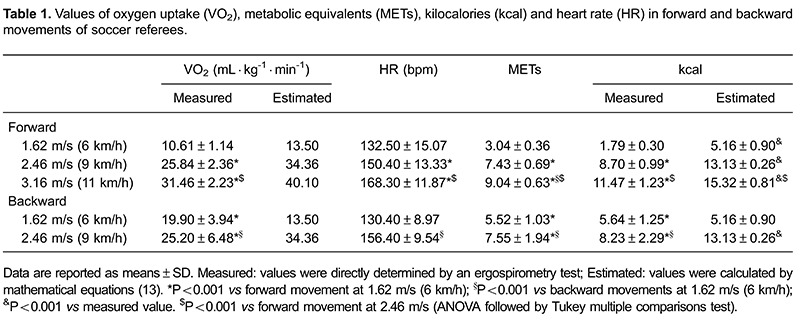

The mechanical efficiency during backward/forward walking and backward/forward jogging was reported as the percentage of net efficiency. The net efficiency of participants during forward and backward walking was 17.98±4.03 and 8.17±2.64%, respectively (P<0.001). In contrast, net efficiency during forward and backward jogging was 6.59±0.83 and 7.63±2.58%, respectively. The low efficiency shown by referees directly reflects the lower VO_2max_ values found.

## Discussion

The aim of this study was to measure the energy expenditure of common motor activities performed by soccer referees during a match in a laboratory setting (walking, jogging, and running), and to compare forward with backward movements. Our results show that backward walking and jogging are not high-intensity activities, as has been commonly reported in the international literature. Furthermore, when energy expenditure was estimated using equations available in the literature, values were significantly overestimated compared to those measured by indirect calorimetry in the laboratory. Results of the ergospirometry test showed a progressively increasing VO_2_ for forward walking, jogging, and running. The mean VO_2_max obtained in this study was moderate, as has been described in the literature for elite European soccer referees (6,20). The literature considers values between 7.5 and 9 kcal to represent moderate energy expenditure (1). Therefore, the only motor activity in our study considered of high intensity (energy expenditure more than 9 kcal) was forward running. VO_2_ during backward walking and jogging was significantly higher than that observed for forward walking, but was similar to that for forward jogging. All backward motor actions showed an energy expenditure of less than 9 kcal; therefore, backward movements could not be considered high-intensity activities in our study. This contradicts the results obtained by Reilly and Bowen (12) who investigated energy consumption in soccer players. The protocol used by these authors was similar to that used in the present study, and they evaluated the motor activities of forward and backward movements at speeds of 5, 7, and 9 km/h. Unfortunately, these authors report energy expenditure in kcal, but no VO_2_ results are reported for each activity. They found a consumption of 12.08±1.18 and 17.06±1.67 kcal for forward and backward movements at 9 km/h, respectively. The difference between the energy expenditure of forward and backward movements at 9 km/h shown in the study of Reilly and Bowen (12) is 4.93 kcal or approximately 40% higher in backward movements. Williford et al. (21) also reported a difference of 15% in both VO_2_ and HR between forward and backward movements at 8 km/h in young tennis players. Based on these studies, backward movements are considered high intensity activities, which call for greater metabolic demand and elevated cardiovascular response. In the present study, we observed similar values of energy expenditure for forward and backward displacements at 2.46 m/s (jogging), a difference of only 5.5%. In addition, the maximum energy expenditure found in the present study was 18.08±6.38 kcal; therefore, these 2 activities use approximately 52% of the maximum energy expenditure from each individual and obviously cannot be regarded as activities with a high energy cost. Differences in measurements and speeds between our study and the studies by Reilly and Bowen (12) and Williford et al. (21) make it difficult to conduct comparisons across these studies. However, it is interesting to note that in the Williford et al. (21) study the difference in energy expenditure between forward and backward movements decreased with an increasing intensity of movement (differences of 28 and 15% for movement at 6 and 8 km/h, respectively), as was observed in our study. Several factors could explain the differences between forward and backward movements: motor patterns (increase of cadence and reduction of stride length during backward movement), differences in muscular power and work output, different types of muscle action, differences in motor unit recruitment, and greater burden on the medial sensorimotor cortices (22 23 24 [Bibr B25] 26). The last two factors should primarily be considered when backward movement is a "new" task, with little or no previous practice carried out (27). All of these factors, and the fluctuation between them, influence the distribution of effort between the aerobic and anaerobic components, hampering the measurement of total energy consumption. However, the increase in speed promotes a reduction in body stability in a similar manner in both types of motor action, which explains the decrease of the difference in energy consumption with increasing intensity (speed) (28 [Bibr B29]). In other words, with the increase in speed, and concomitant increased demand of the anaerobic system, there is a reduction in the consumption of oxygen.

Energy expenditure of an athlete during physical activity can be predicted by mathematical equations. In the present experimental protocol we observed that these equations overestimated energy expenditure in at least 35%. In a previous study, the energy expenditure of soccer referees during official matches was estimated from equations that take into account the time spent performing each motor activities (16). By using published equations, Da Silva and Rodriguez-Aãez (16) reported a total energy expenditure of 740.42 and 494.64 kcal for referees and assistant referees, respectively. The referees consumed a mean O_2_ of 16.17, 33.08, and 41.46 mL·kg^-1^·min^-1^ for walking, jogging, and running activities, respectively. Furthermore, when VO_2_ was transformed into kcal and the values for these 3 motor activities were combined, a total of 705.48 kcal consumption during a 90 min match was obtained. By using mathematical equations, which combine the time spent on motor activities with the VO_2_ values, we found a total of 469.76 kcal (walking, 225.27 kcal; jogging, 135.28 kcal; running, 110.20 kcal). Thus, in our study, the equation for VO_2_ overestimated the energy expenditure in the participants. This observation could be owing to differences in the weight of the subjects (88 kg in the study by Da Silva and Rodriguez-Aãez (16) and 77.5 kg in the present study). However, when the same routine was performed by assistant referees from the study by Da Silva and Rodriguez-Aãez, who showed an average weight of 77 kg, a similar pattern was observed (walking, 328.05 *vs* 215.60 kcal; jogging, 42.02 *vs* 25.43 kcal; running, 18.62 *vs* 13.40 kcal). The total energy expenditure estimated from equations in the Da Silva and Rodriguez-Aãez study was 388.69 kcal, which was considerably higher than that found in our study by using VO_2_ values (254.44 kcal). Another possible explanation for the difference in energy expenditure between the studies could be the different duration of each activity.

Da Silva et al. (13) estimated the energy expenditure of soccer referees during official matches in METs and in kcal. These authors reported a total energy expenditure of 734.7±65 kcal, made up of 266.05, 256.25, 94.68, and 85.57 kcal for walking, jogging, running, and backward movement, respectively. Unfortunately, our study did not evaluate the individual energy expenditure for sprinting, which hampered the comparison of total consumption during the match between the studies.

One way to explain differences in individual energy expenditures is through exercise efficiency. The net efficiency reflects how efficiently our organism can transduce oxygen consumption into effective work. Several mathematical equations have been developed to describe individual efficiency during a physical activity (2,18). It is known that many factors influence energy expenditure both at rest and during activity, such as the intensity of the activity, movement speed, and the different composition of motor units in each muscle, amongst others (2). Diverse studies have found that a marathon runner has an average efficiency of 20 to 25% and that swimming is the least efficient activity, promoting an efficiency of about 7% [2,18,29]. Powers and Howley (2) reported that mechanical efficiency is inversely proportional to intensity; in other words, the higher the intensity, the lower the efficiency. This is evident when looking at the data for net efficiency of forward and backward movements at 9 km/h (jogging). Furthermore, we observed that the highest efficiency was obtained in forward movements at 6 km/h (walking) (17.98±4.03%). During this motor activity, referees presented a mean energy expenditure of only 3.04±0.36 METs and 1.79±0.30 kcal, just above resting values. One hypothesis that could explain the differences in mechanical efficiency is the professional experience as referees, and body fat percentage. However, no correlations between professional experience or body fat % and VO_2_ consumption in forward or backward walking/jogging are reported in the literature. Moreover, some papers have reported differences among the metabolic systems used, and in test/re-test measurement evaluations (30,31). These factors could explain the high variability of data published for backward walking and jogging.

The main limitation of this study is that referees were evaluated at specific workloads, which may not correspond to actual speeds when walking/jogging in a match situation. Also, in a lab setting, environmental factors like temperature, humidity, precipitation, wind, etc, can be controlled, which could be very different to a match situation. However, these results contribute to the knowledge on the energetic expenditure during training sessions, and provide information that may be useful in body weight control during the season.

In summary, activities such as backward walking and jogging should not be considered as high-intensity activities, based in ergospirometry tests results. When calculated using equations available in the literature, energy expenditure can be overestimated and produce errors when prescribing diets. A diet plan based on overestimated energy consumption may cause greater calorie intake than required, which could contribute to the high percentage of body fat commonly found in referees (32 33-34). Future research should focus in field measurements of energy expenditure and dietetic strategies specific to refereeing.
